# Supplementary material for: Novel strains of Campylobacter cause diarrheal outbreak in Rhesus macaques (Macaca mulatta) of Kathmandu Valley
Source: PLoS One. 2023 Mar 1;18(3):e0270778. doi: 10.1371/journal.pone.0270778 (PMC9977009; doi:10.1371/journal.pone.0270778)
Supplement: S1 File — (DOCX) [file pone.0270778.s001.docx]

**Supplementary Table 1:** Location of collected diarhhea fecal samples of Rhesus macaque

| **S.N.** | **Sample Code** | **Date** | **Lattitude (oN)** | **Longitude (oE)** |
| --- | --- | --- | --- | --- |
| **1** | Fe-SA002 | 29/06/2018 | 27.71582 | 85.284767 |
| **2** | Fe-SA003 | 29/06/2018 | 27.71587 | 85.284813 |
| **3** | Fe-SA004 | 29/06/2018 | 27.71587 | 85.284816 |
| **4** | Fe-SA005 | 29/06/2018 | 27.71573 | 85.284935 |
| **5** | Fe-SA006 | 29/06/2018 | 27.71579 | 85.285133 |
| **6** | Fe-SA007 | 29/06/2018 | 27.71571 | 85.285363 |
| **7** | Fe-SA008 | 29/06/2018 | 27.71582 | 85.2851 |
| **8** | Fe-SA009 | 29/06/2018 | 27.71587 | 85.285623 |
| **9** | Fe-SA010 | 29/06/2018 | 27.71584 | 85.285666 |
| **10** | Fe-SA011 | 29/06/2018 | 27.71583 | 85.285673 |
| **11** | Fe-SB008 | 29/06/2018 | 27.71535 | 85.288085 |
| **12** | Fe-SB009 | 29/06/2018 | 27.7154 | 85.288145 |
| **13** | Fe-SB010 | 29/06/2018 | 27.71543 | 85.288179 |
| **14** | Fe-SB011 | 29/06/2018 | 27.7155 | 85.28813 |
| **15** | Fe-SC001 | 28/06/2018 | 27.71544 | 85.28955 |
| **16** | Fe-SC002 | 28/06/2018 | 27.7152 | 85.28954 |
| **17** | Fe-SC003 | 28/06/2018 | 27.71545 | 85.28952 |
| **18** | Fe-SC004 | 28/06/2018 | 27.71525 | 85.2897 |
| **19** | Fe-SC005 | 28/06/2018 | 27.71519 | 85.28973 |
| **20** | Fe-SC006 | 28/06/2018 | 27.71479 | 85. 29015 |
| **21** | Fe-SC007 | 28/06/2018 | 27.71477 | 85.29018 |
| **22** | Fe-SC008 | 28/06/2018 | 27.7149 | 85.29024 |
| **23** | Fe-SC009 | 28/06/2018 | 27.71528 | 85.29018 |
| **24** | Fe-SC010 | 28/06/2018 | 27.71528 | 85.29014 |
| **25** | Fe-SC011 | 28/06/2018 | 27.71522 | 85.29011 |
| **26** | Fe-SD001 | 28/06/2018 | 27.7149 | 85.29143 |
| **27** | Fe-SD004 | 28/06/2018 | 27.71438 | 85.29279 |
| **28** | Fe-SD006 | 28/06/2018 | 27.71429 | 85.29312 |
| **29** | Fe-SD007 | 28/06/2018 | 27.71423 | 85.29309 |
| **30** | Fe-SD008 | 28/06/2018 | 27.71428 | 85.29316 |
| **31** | Fe-SE001 | 29/06/2018 | 27.71718 | 85.28647 |
| **32** | Fe-SE002 | 29/06/2018 | 27.71771 | 85.2895 |
| **33** | Fe-SE003 | 29/06/2018 | 27.71762 | 85.28975 |
| **34** | Fe-SE007 | 29/06/2018 | 27.71587 | 85.28971 |
| **35** | Fe-SE009 | 29/06/2018 | 27.7158 | 85.28955 |
| **36** | Fe-PA003 | 1/7/2018 | 27.70954 | 85.346222 |
| **37** | Fe-PA004 | 1/7/2018 | 27.71 | 85.346489 |
| **38** | Fe-PA005 | 1/7/2018 | 27.71005 | 85.34684 |
| **39** | Fe-PA010 | 1/7/2018 | 27.70988 | 85.348701 |
| **40** | Fe-PA011 | 1/7/2018 | 27.70996 | 85.348526 |
| **41** | Fe-PB001 | 30/06/2018 | 27.70973 | 85.350664 |
| **42** | Fe-PB002 | 30/06/2018 | 27.70966 | 85.350422 |
| **43** | Fe-PB005 | 30/06/2018 | 27.70958 | 85.349586 |
| **44** | Fe-PB009 | 30/06/2018 | 27.70958 | 85.349089 |
| **45** | Fe-PB011 | 30/06/2018 | 27.70967 | 85.349269 |
| **46** | Fe-PC003 | 30/06/2018 | 27.71019 | 85.351018 |
| **47** | Fe-PC006 | 30/06/2018 | 27.70991 | 85.35115 |
| **48** | Fe-PC007 | 30/06/2018 | 27.71 | 85.351174 |
| **49** | Fe-PC009 | 30/06/2018 | 27.71015 | 85.351286 |
| **50** | Fe-PC011 | 30/06/2018 | 27.71023 | 85.35111 |
| **51** | Fe-PD001 | 30/06/2018 | 27.71251 | 85.35057 |
| **52** | Fe-PD002 | 30/06/2018 | 27.71257 | 85.35052 |
| **53** | Fe-PD003 | 30/06/2018 | 27.71257 | 85.35052 |
| **54** | Fe-PD004 | 30/06/2018 | 27.7126 | 85.35051 |
| **55** | Fe-PD005 | 30/06/2018 | 27.71287 | 85.35027 |
| **56** | Fe-PD006 | 30/06/2018 | 27.71291 | 85.35022 |
| **57** | Fe-PD007 | 30/06/2018 | 27.7129 | 85.35012 |
| **58** | Fe-PD008 | 30/06/2018 | 27.713 | 85.35003 |
| **59** | Fe-PD009 | 30/06/2018 | 27.71297 | 85.34999 |
| **60** | Fe-PD010 | 30/06/2018 | 27.71292 | 85.34989 |
| **61** | Fe-PD011 | 30/06/2018 | 27.71293 | 85.35006 |
| **62** | Fe-PE002 | 1/7/2018 | 27.70921 | 85.34337 |
| **63** | Fe-PE005 | 1/7/2018 | 27.70909 | 85.34336 |
| **64** | Fe-PE006 | 1/7/2018 | 27.70908 | 85.34322 |
| **65** | Fe-PE007 | 1/7/2018 | 27.70904 | 85.34319 |
| **66** | Fe-PE010 | 1/7/2018 | 27.70825 | 85.34273 |
| **67** | Fe-PE011 | 1/7/2018 | 27.70826 | 85.34274 |

**Supplementary Table 2:** Reference sequences 16S rRNA used in this study

| **S.N.** | **Species** | **Host** | **Country** | **Genbank Accession No** |
| --- | --- | --- | --- | --- |
| 1 | Campylobacter hominis | Human | England | AJ251584.1 |
| 2 | Campylobacter hominis | Human | England | AF062492.1 |
| 3 | Campylobacter sputorum | Ovine | Sweden | AF550635 |
| 4 | Campylobacter sputorum | Human | Canada | AF550636.1 |
| 5 | Campylobacter sputorum | Bovine | Austria | AF550639.1 |
| 6 | Campylobacter corcagiensis | Macaca silenus | NA | NR_133992.1 |
| 7 | Campylobacter canadensis | Whooping Crane | Canada | EF621894 |
| 8 | Campylobacter ureolyticus | NA | NA | NR_118654.1 |
| 9 | Campylobacter ureolyticus | Human | Germany | NR_117766.1 |
| 10 | Campylobacter gracilis | Human | USA | AF550656.1 |
| 11 | Campylobacter gracilis | Human | NA | L04320.1 |
| 12 | Campylobacter rectus | Human | Sweden | AF550659.1 |
| 13 | Campylobacter showae | Human | USA | AF550655.1 |
| 14 | Campylobacter showae | Human | Sweden | L06975.1 |
| 15 | Campylobacter curvus | Human | USA | AF550650.1 |
| 16 | Campylobacter curvus | Human | Belgium | AF550652.1 |
| 17 | Campylobacter concisus | Human | Sweden | AF550653.1 |
| 18 | Campylobacter concisus | Human | Belgium | AF550654.1 |
| 19 | Campylobacter mucosalis | Porcrine | Scotland | AF550660.1 |
| 20 | Campylobacter mucosalis | Porcrine | Scotland | AF550663.1 |
| 21 | Campylobacter mucosalis | Porcrine | Scotland | AF550662.1 |
| 22 | Campylobacter iguaniorum | Iguana | NA | KF425532.1 |
| 23 | Campylobacter iguaniorum | Iguana | NA | KF425533.1 |
| 24 | Campylobacter iguaniorum | Iguana | NA | KF787891.1 |
| 25 | Campylobacter iguaniorum | Iguana | NA | KF787893.1 |
| 26 | Campylobacter fetus subsp. fetus | Sheep | Turkey | MK806573 |
| 27 | Campylobacter fetus subsp. fetus | Human | Japan | AB301966 |
| 28 | Campylobacter fetus subsp. venerealis | Bull | NA | DQ174133 |
| 29 | Campylobacter fetus subsp. venerealis | Cow | NA | DQ174131 |
| 30 | Campylobacter fetus subsp. fetus | Human | NA | DQ174130 |
| 31 | Campylobacter hyointestinalis subsp. hyointestinalis | Deer | Japan | LC388476 |
| 32 | Campylobacter hyointestinalis subsp. hyointestinalis | Human | NA | DQ174179 |
| 33 | Campylobacter hyointestinalis subsp. hyointestinalis | Sheep | Spain | HQ628643 |
| 34 | Campylobacter hyointestinalis subsp. hyointestinalis | Porcine | NA | AF097691 |
| 35 | Campylobacter lanienae | Pig | Hungary | HM462470 |
| 36 | Campylobacter lanienae | Wild Boar | Japan | LC388449 |
| 37 | Campylobacter lanienae | Pig | Ecuador | KU362563 |
| 38 | Campylobacter lanienae | Human | NA | AF043424 |
| 39 | Campylobacter hyointestinalis subsp. lawsonii | Pig | NA | DQ195237 |
| 40 | Campylobacter hyointestinalis subsp. lawsonii | Swine | Spain | HQ628645 |
| 41 | Campylobacter hyointestinalis subsp. lawsonii | Porcine | NA | AF097684 |
| 42 | Campylobacter hyointestinalis subsp. lawsonii | Porcine | NA | AF097683 |
| 43 | Campylobacter hyointestinalis subsp. lawsonii | Goat | India | MN203692 |
| 44 | Campylobacter hyointestinalis | Human | India | MN203681 |
| 45 | Unknown Campylobacter spp. | Rhesus Monkey | Nepal | MZ068107 |
| 46 | Unknown Campylobacter spp. | Rhesus Monkey | Nepal | MZ068108 |
| 47 | Unknown Campylobacter spp. | Rhesus Monkey | Nepal | MZ068109 |
| 48 | Unknown Campylobacter spp. | Horse | NA | EF564132 |
| 49 | Unknown Campylobacter spp. | Horse | NA | EF564134 |
| 50 | Unknown Campylobacter spp. | Horse | NA | EF564135 |
| 51 | Unknown Campylobacter spp. | Rhesus Monkey | Nepal | MZ068100 |
| 52 | Unknown Campylobacter spp. | Rhesus Monkey | Nepal | MZ068101 |
| 53 | Unknown Campylobacter spp. | Human | Tanzania | MT131157 |
| 54 | Unknown Campylobacter spp. | Human | Tanzania | MT130980 |
| 55 | Unknown Campylobacter spp. | Human | Tanzania | MT130976 |
| 56 | Unknown Campylobacter spp. | Rhesus Monkey | Nepal | MZ068102 |
| 57 | Candidatus Campylobacter infans | Human | Netherlands | CP049075 |
| 58 | Campylobacter hyointestinalis subsp. lawsonii | Sheep | India | MN203694 |
| 59 | Campylobacter hyointestinalis subsp. lawsonii | Human | India | MN203696 |
| 60 | Unknown Campylobacter spp. | Rhesus Monkey | Nepal | MZ068103 |
| 61 | Unknown Campylobacter spp. | Rhesus Monkey | Nepal | MZ068104 |
| 62 | Unknown Campylobacter spp. | Rhesus Monkey | Nepal | MZ068105 |
| 63 | Unknown Campylobacter spp. | Rhesus Monkey | Nepal | MZ068106 |
| 64 | Campylobacter helveticus | Cat | NA | DQ174163 |
| 65 | Campylobacter vulpis | Red Fox | Italy | KU855032 |
| 66 | Campylobacter vulpis | Red Fox | Italy | KU855039 |
| 67 | Campylobacter vulpis | Red Fox | Italy | KU855043 |
| 68 | Campylobacter upsaliensis | Canine | NA | AF550642 |
| 69 | Unknown Campylobacter spp. | Bush Dog | USA | EU463358 |
| 70 | Campylobacter upsaliensis | Human | NA | DQ174160 |
| 71 | Campylobacter upsaliensis | Dog | NA | DQ174157 |
| 72 | Campylobacter upsaliensis | Human | NA | AF550640 |
| 73 | Campylobacter upsaliensis | Dog | NA | JX912527 |
| 74 | Campylobacter upsaliensis | Human | Japan | AB980278 |
| 75 | Unknown Campylobacter spp. | Yunnan Snub Nosed Monkey | China | GQ451185 |
| 76 | Campylobacter troglodytis | Chimpanzee | Tanzania | EU559330 |
| 77 | Campylobacter troglodytis | Chimpanzee | Tanzania | HQ864829 |
| 78 | Campylobacter upsaliensis | Human | India | MN203690 |
| 79 | Unknown Campylobacter spp. | Human | Tanzania | MT130985 |
| 80 | Unknown Campylobacter spp. | Rhesus Monkey | Nepal | MZ068110 |
| 81 | Unknown Campylobacter spp. | Rhesus Monkey | Nepal | MZ068111 |
| 82 | Unknown Campylobacter spp. | Rhesus Monkey | Nepal | MZ068112 |
| 83 | Campylobacter cuniculorum | Rabbit | Italy | DQ400345 |
| 84 | Campylobacter cuniculorum | Rabbit | Italy | EU636816 |
| 85 | Unknown Campylobacter spp. | Red Kangaroo | USA | EU461051 |
| 86 | Campylobacter jejuni subsp. jejuni | Human | India | MN203682 |
| 87 | Campylobacter coli | Human | Belgium | AF550620.1 |
| 88 | Campylobacter lari | Human | Canada | AF550633.1 |
| 89 | Campylobacter coli | Human | Australia | AF550622.1 |
| 90 | Campylobacter jejuni subsp. jejuni | Human | NA | DQ174142 |
| 91 | Campylobacter coli | Wild Boar | Japan | LC388445 |
| 92 | Campylobacter coli | Broiler | Sweden | EU127530 |
| 93 | Campylobacter coli | Cow | Turkey | KT225284 |
| 94 | Campylobacter jejuni subsp. jejuni | Human | NA | AF550626 |
| 95 | Campylobacter coli | Human | Japan | AB587647 |
| 96 | Campylobacter coli | Dog | India | MK156110 |
| 97 | Campylobacter jejuni subsp. jejuni | Crow | India | MK156114 |
| 98 | Campylobacter coli | Goat | NA | KJ777705 |
| 99 | Campylobacter jejuni subsp. jejuni | Pig | India | MK156115 |
| 100 | Campylobacter jejuni subsp. doylei | Human | NA | Y19244 |
| 101 | Campylobacter upsaliensis | Canine | Spain | MT453978 |
| 102 | Campylobacter jejuni subsp. jejuni | Broiler | Sweden | EU127548 |
| 103 | Campylobacter jejuni subsp. doylei | Human | NA | DQ174144 |
| 104 | Campylobacter jejuni subsp. jejuni | Bovine | NA | AF372091 |
| 105 | Campylobacter coli | Water | NA | DQ174137 |
| 106 | Campylobacter troglodytis | Cotton Topped Tamarin | USA | EU559331 |
| 107 | Campylobacter helveticus | Feline | NA | AF550647 |
| 108 | Campylobacter helveticus | Feline | NA | AF550646 |
| 109 | Campylobacter helveticus | Cat | NA | DQ174161 |
| 110 | Campylobacter helveticus | Dog | NA | DQ174164 |
| 111 | Unknown Campylobacter spp. | Prairie Dog | USA | HM211850 |
| 112 | Campylobacter lari | Human | Belgium | AF550631.1 |
| 113 | Campylobacter lari | Avian | NA | L04316.1 |
