# Supplementary material for: Novel strains of Campylobacter cause diarrheal outbreak in Rhesus macaques (Macaca mulatta) of Kathmandu Valley
Source: PLoS One. 2023 Mar 1;18(3):e0270778. doi: 10.1371/journal.pone.0270778 (PMC9977009; doi:10.1371/journal.pone.0270778)
Supplement: S2 File — (DOCX) [file pone.0270778.s002.docx]

**Supplementary Information**
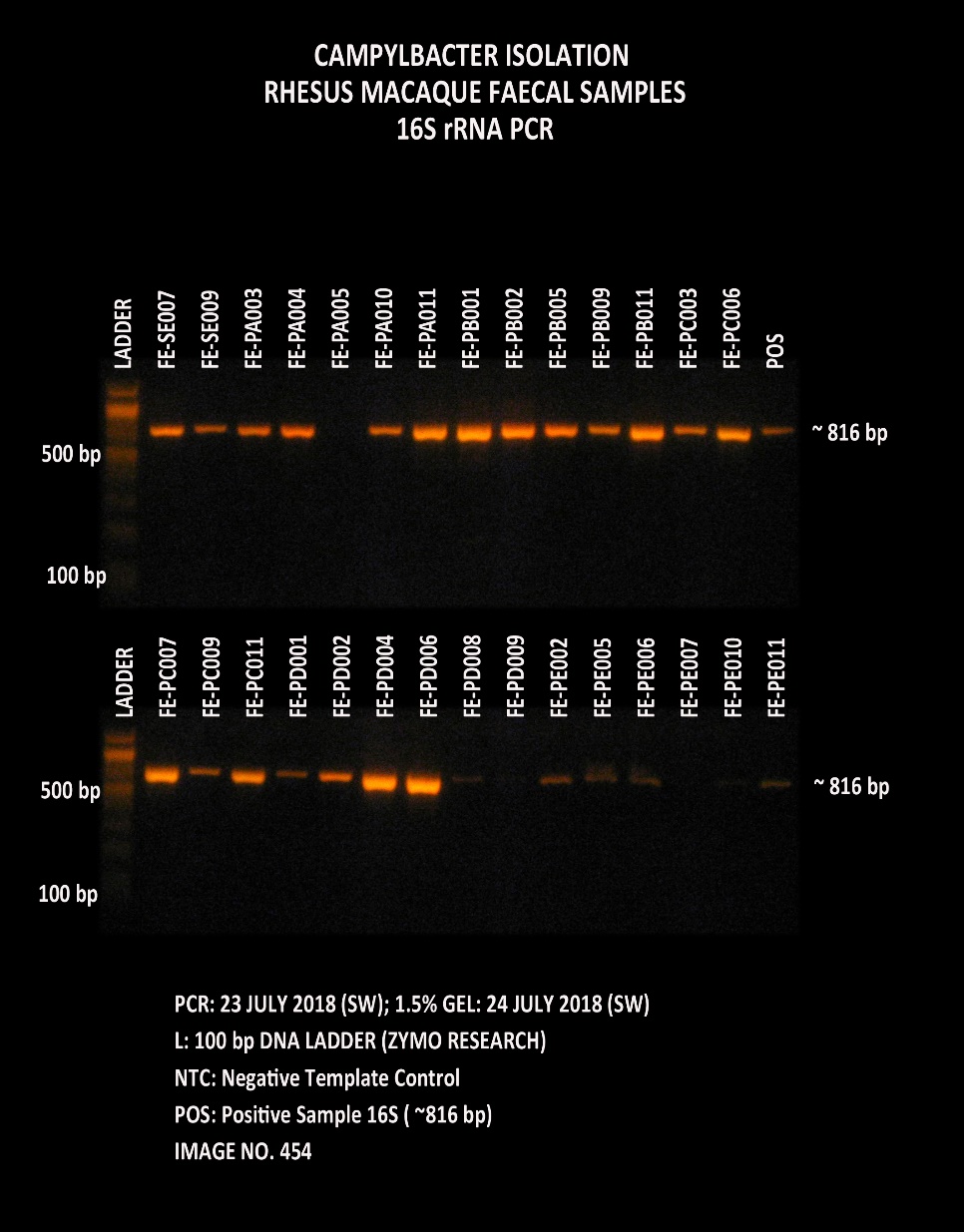

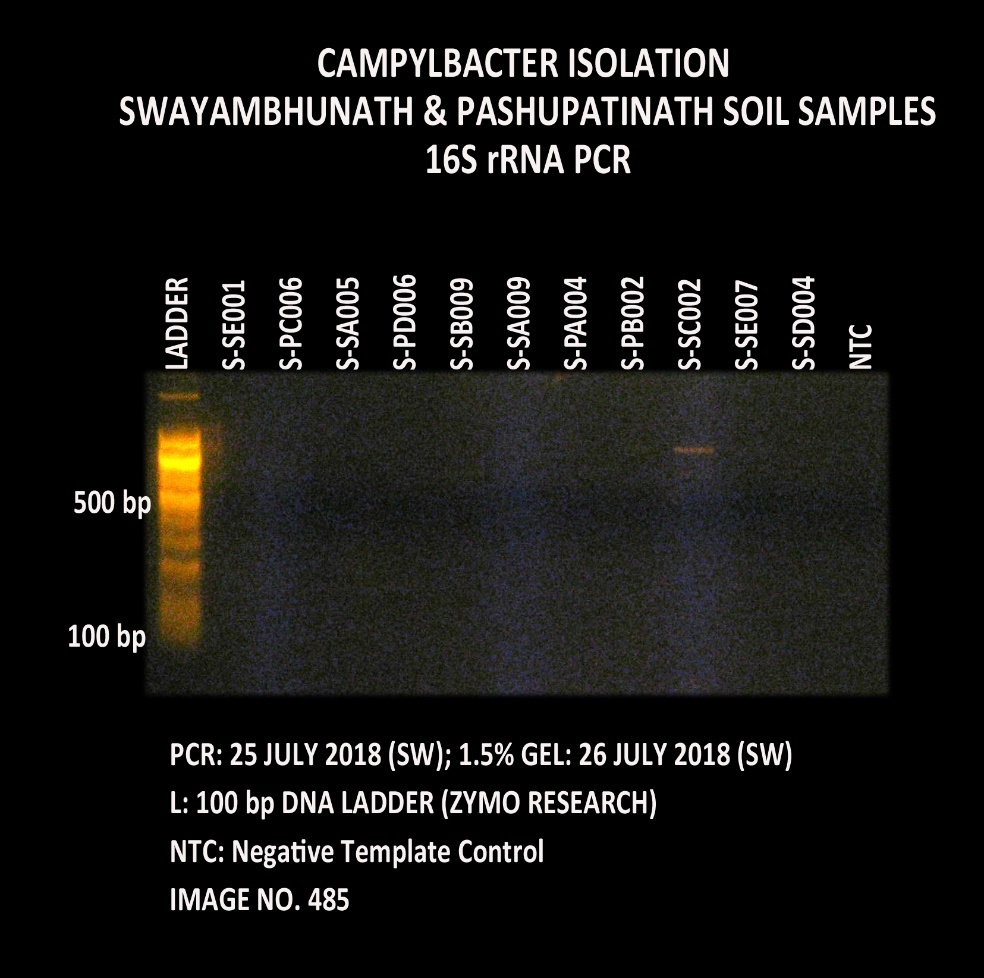

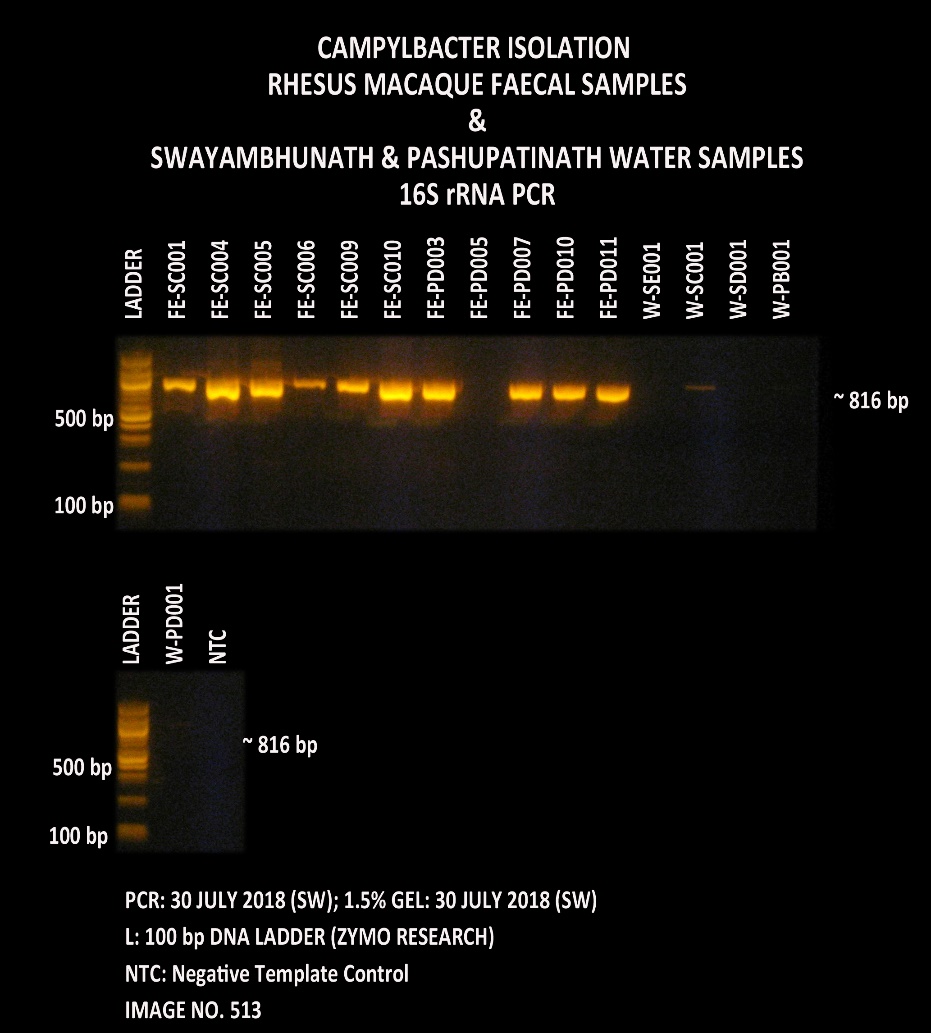


a.

b.

c.

**Supplementary Figure 1:** 16S rRNA based PCR detection of a) Campylobacter sp. from 27 Rhesus macaque fecal samples. b) Campylobacter sp. from soil samples. c) Campylobacter sp. from water samples.


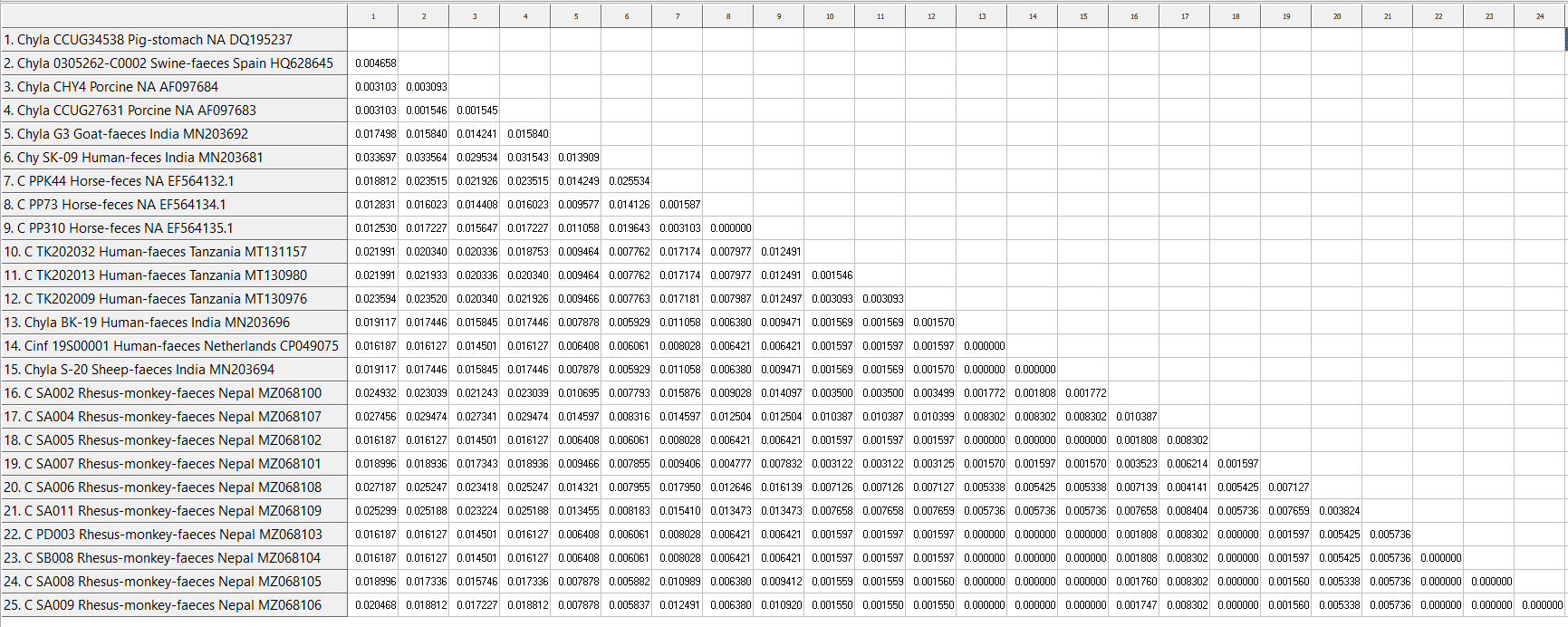


**Supplementary Figure 2:** Kimura-2 parameter pairwise distance calculation of clade-1 isolates compared to isolates of *C. hyointestinalis subsp. lawsonii* and *Candidatus C. infans*

**Supplementary Figure 3:** Kimura-2 parameter pairwise distance calculation of clade-2 with isolates of *C. vulpis, C. upsaliensis, C. troglodytes* & *C. helveticus*


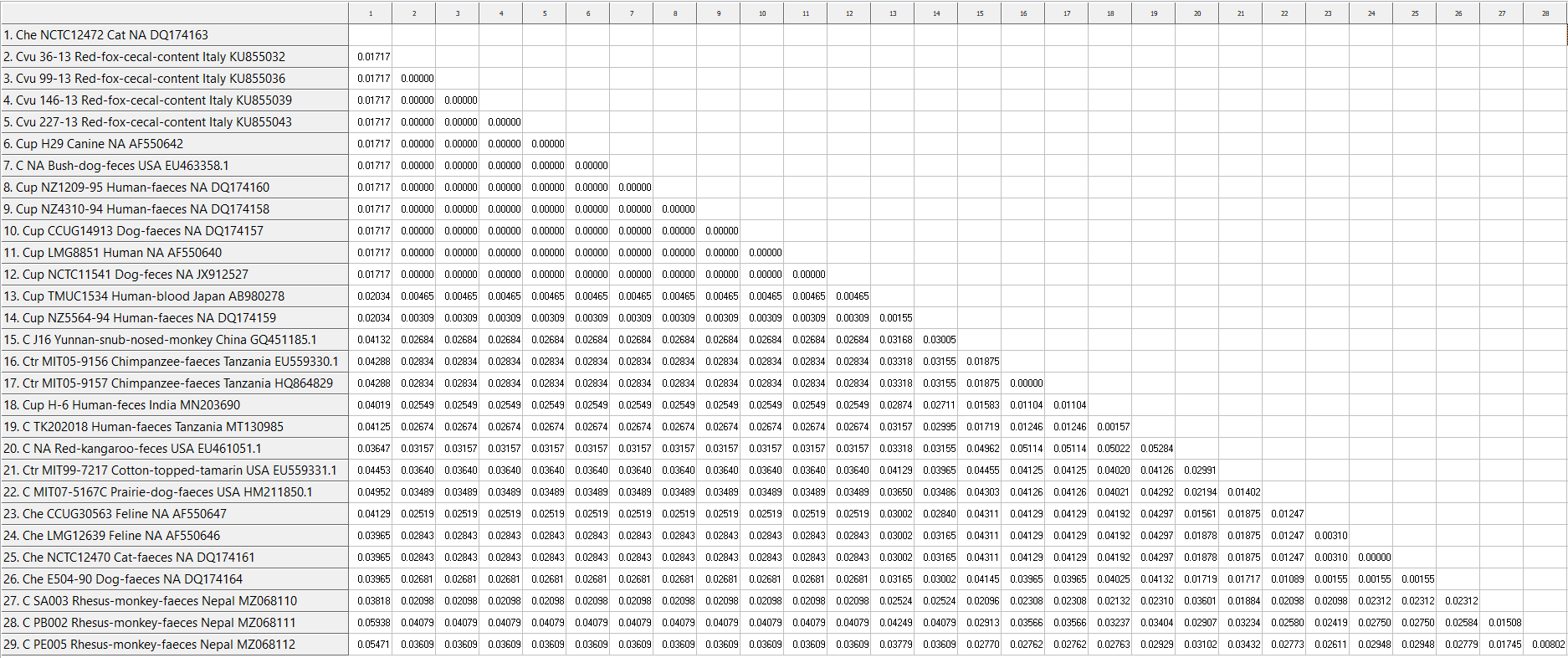


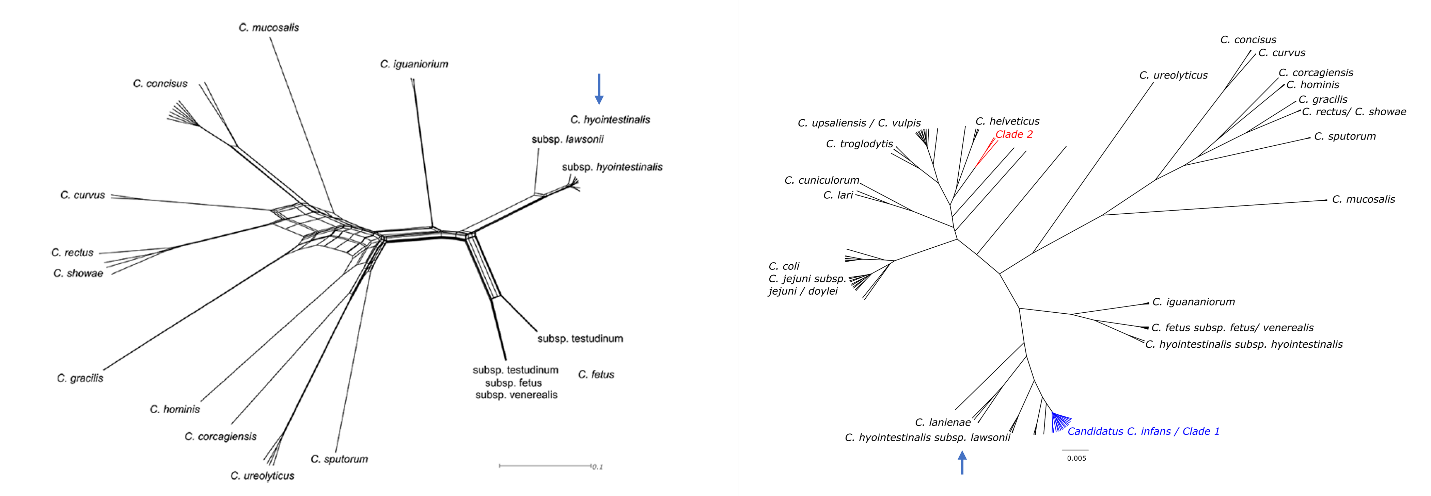


**Supplementary Figure 4:** Comparison of core genome alignment network for non-thermophilic *Campylobacter* species (right) from D. A. Wilkinson, et.al., (2018) and partial 16srRNA gene sequences obtained in this study. Phylogenetics prepared by using BEAST v 2.6.4 (left). Arrow indicates clade from same species (for ease of comparison).
