# Supplementary material for: Novel strains of Campylobacter cause diarrheal outbreak in Rhesus macaques (Macaca mulatta) of Kathmandu Valley
Source: PLoS One. 2023 Mar 1;18(3):e0270778. doi: 10.1371/journal.pone.0270778 (PMC9977009; doi:10.1371/journal.pone.0270778)

Raw image of Figure 2

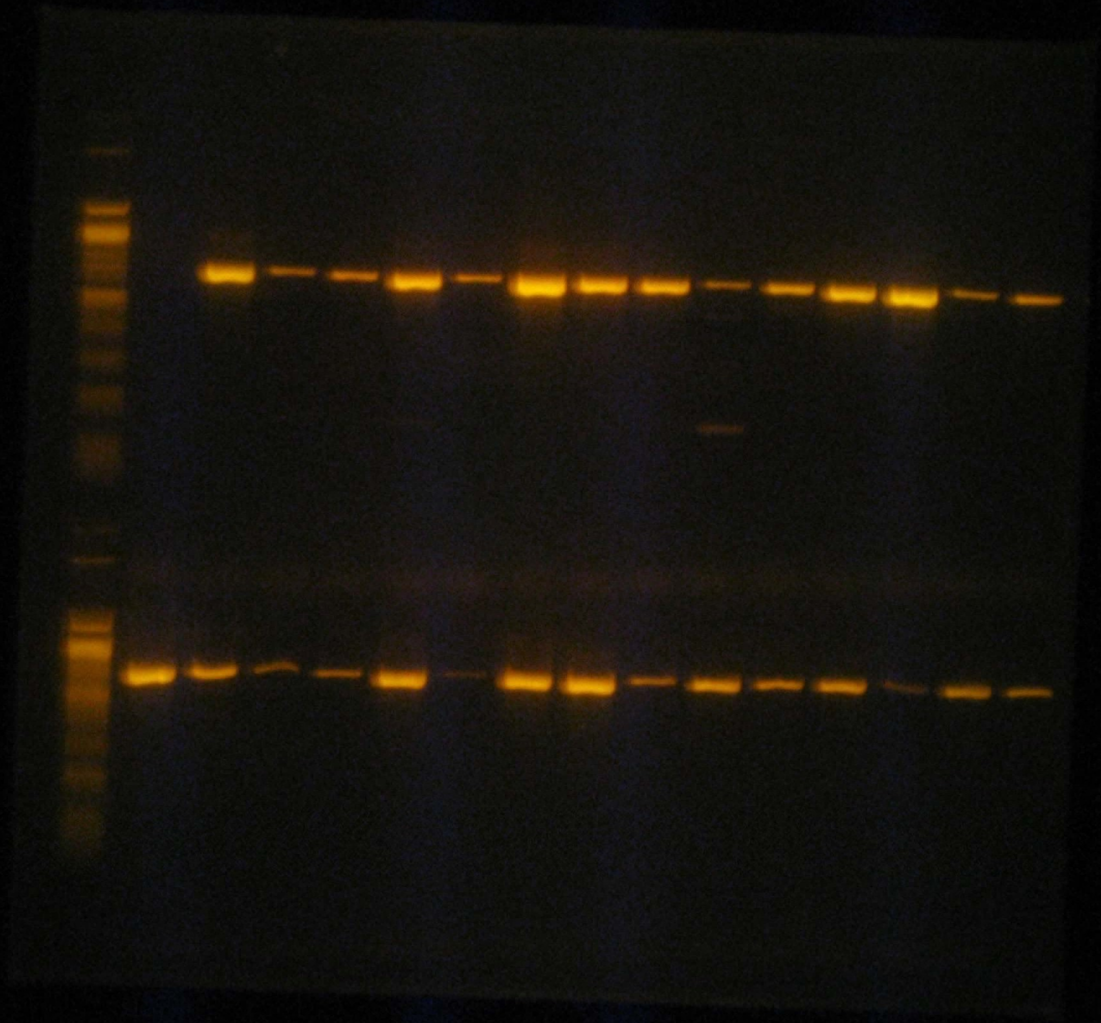

Raw image of Supplementary 1b -Campylobacter screening on Soil sample

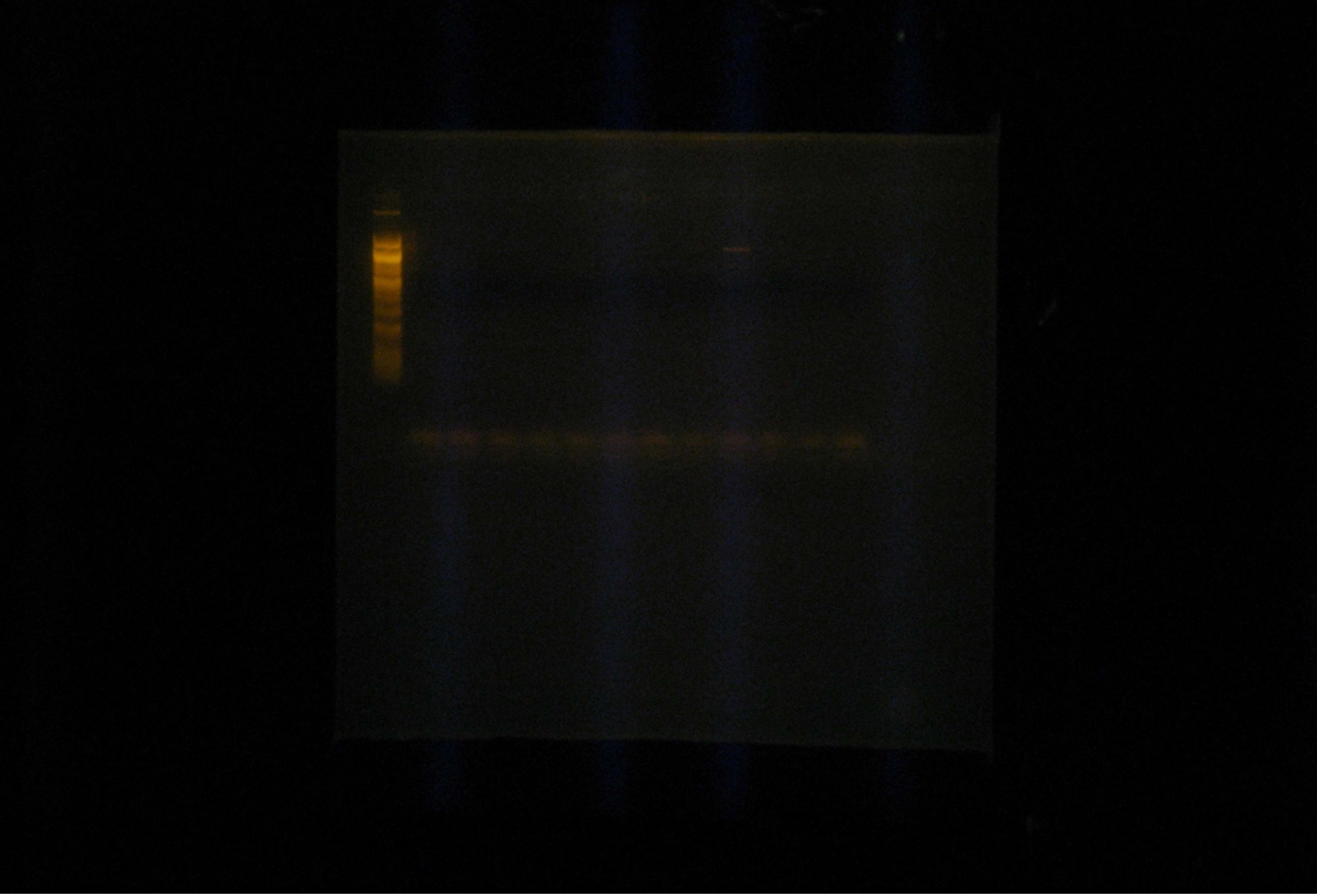

Raw image of supplementary figure 1a

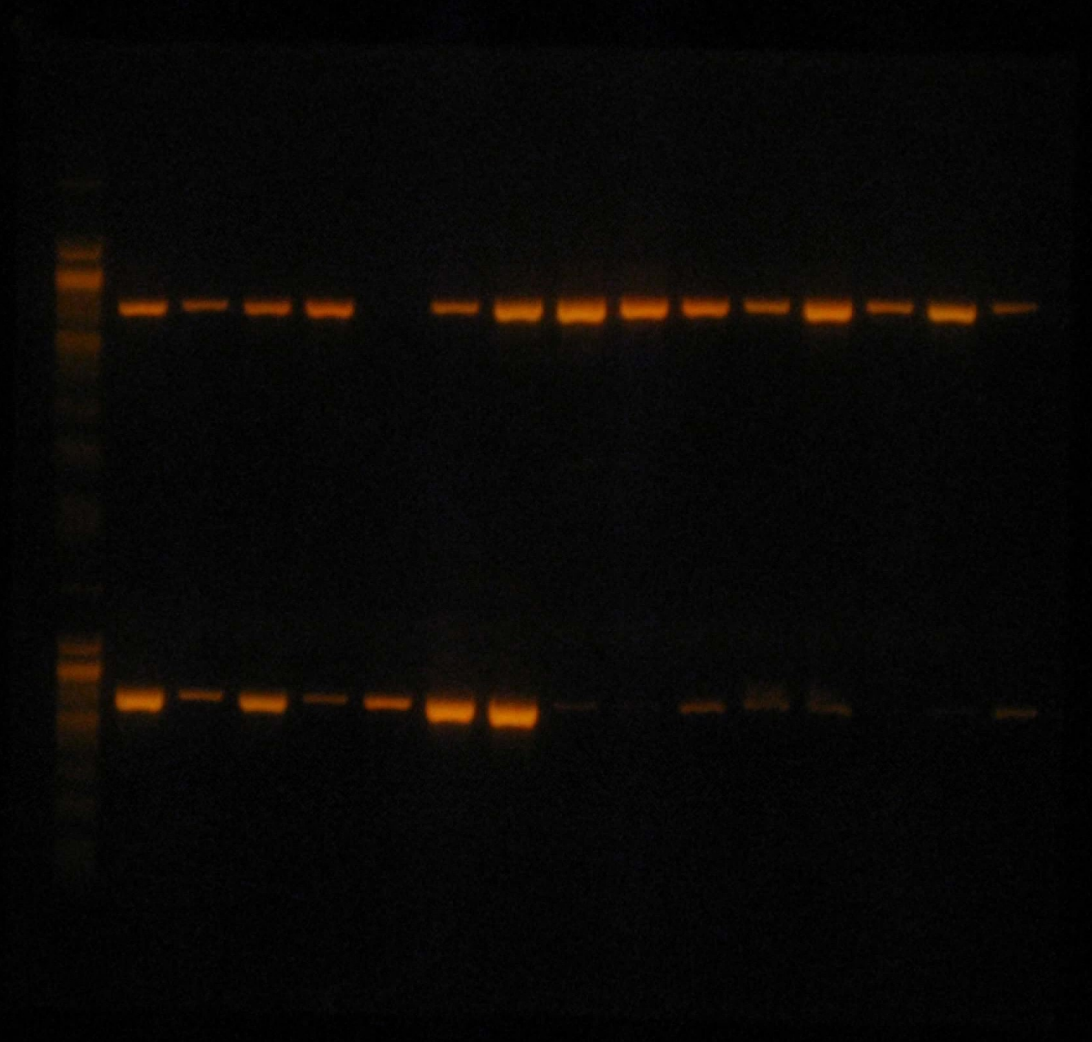

Raw image of supplementary fig 1c- campylobacter screening fecal and water sample.

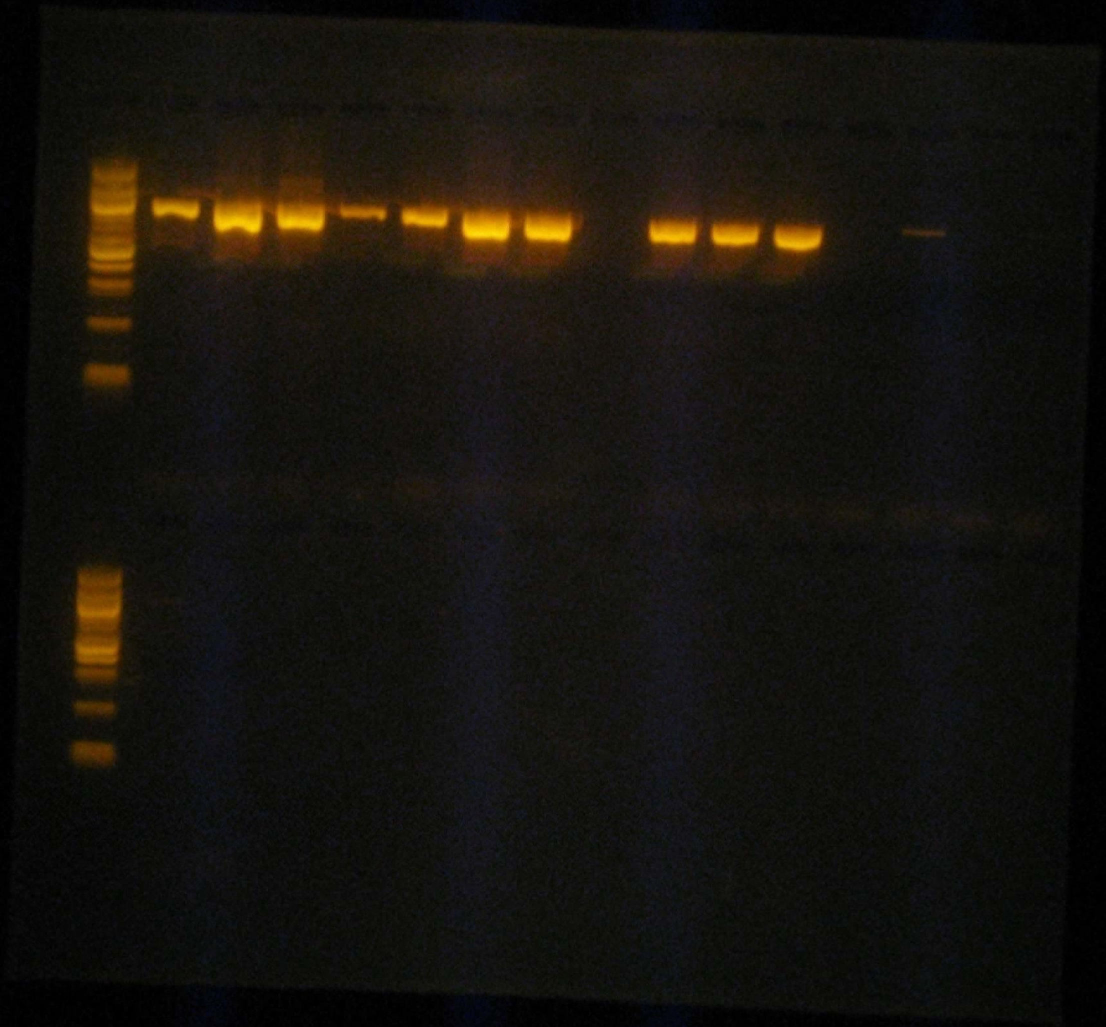

Supplement: S1 Raw images — (PDF) [file pone.0270778.s003.pdf]
