# Supplementary material for: Novel strains of Campylobacter cause diarrheal outbreak in Rhesus macaques (Macaca mulatta) of Kathmandu Valley
Source: PLoS One. 2023 Mar 1;18(3):e0270778. doi: 10.1371/journal.pone.0270778 (PMC9977009; doi:10.1371/journal.pone.0270778)
Supplement: Attachment — Submitted filename: rebutal_letter.docx [file pone.0270778.s005.docx]

**CENTER FOR MOLECULAR DYNAMICS NEPAL**

[**www.cmdn.org**](http://www.cmdn.org)

**( PAN 302834428 SWC 24292)**

November 13, 2022

To,

The Editor

PLOS ONE

RE: Submission of our study “**Novel strains of Campylobacter cause diarrheal outbreak in Rhesus macaques (*Macaca mulatta*) of Kathmandu Valley**”.

Dear Sir/Madam,

Greetings from the Center for Molecular Dynamics Nepal!

We are re-submitting our manuscript titled- “*Novel strains of Campylobacter cause diarrheal outbreak in Rhesus macaques (Macaca mulatta) of Kathmandu Valley*” with all the edits and changes as per reviewer’s comment.

Also, figures with probable copy right issues has been resolved with copy right link in the caption section from openstreet map which complies with the journal’s requirement (Openstreet map is listed in Plos one journal website as an approved map site to be used- <https://journals.plos.org/plosone/s/licenses-and-copyright>) and all the gel image has been added ( supplementary figure has been replaced with new one and raw images has been added as well in pdf format).

All these changes and additions are marked with “track changes” in its newer format. Along with this cover letter,

please find response to review letter responding to reviewers’ comments and suggestions. We believe these changes

substantially improved the manuscript and made it publication worthy.

We would like to thank the reviewers for their valuable feedback.

Sincerely,

Dibesh Karmacharya, Ph.D

Chairman/Executive Director

CMDN

5^th^ Floor Swaraj Sadan, Prasuti Griha Marg, Thapathali-11, Kathmandu

Ph +977 1 4251590; email: [info@cmdn.org](mailto:info@cmdn.org)

| Reviewer #1: | This study reports the novel strains of Campylobacter causing diarrhoeal outbreaks in monkeys in two holy sites of Kathmandu valley. | | |
| --- | --- | --- | --- |
|  |  |  |  |
| Major Comment | 1 | In abstract, the authors have mentioned “Opportunistic diarrheal samples of monkeys were analyzed to ………………” It is better to write diarrhoeal samples only instead of opportunistic samples. | It has been replaced with Diarrheal samples. |
| Major Comment | 2 | In abstract, your result highlights the detection of Campylobacter in soil and water samples but methodology part is missing. Please mention about collection and processing of soil and water samples | We used same protocol to process samples (soil, water), we added the information in line 107. |
| Major Comment | 3 | In methods section, line 99, the authors have mentioned water samples were collected form ponds. In contrast to this, in line 102, they have mentioned 5 water samples from river and drinking water sources. Please write exactly which samples you have collected. Also elaborate on the collection and transportation of water samples. | We have now clarified the water sample collection process (pond at Swoyambhu /river at Pashupatinath) in line 99 and (pond/river) in line 99. |
| Major Comment | 4 | In figure 1, please show the sample collection sites for water and soil samples. | This has now been updated. |
| Minor Comment | 5 | Please correct the grammatical errors in the text such as **Line 81: Please rearrange the sentence.** | The sentence has been restructured. |
| Minor Comment | 6 | **Line 100: Please mention the name of the laboratory instead of our lab.** | Our lab has been replaced with the Intrepid Nepal Lab. In line 101. |
| Minor Comment | 7 | **Line 111: Please write the symbol** **µ instead of u** | u has been replaced with symbol µ. |
| Minor Comment | 8 | **Line 111-113: Please complete the sentence “ The cycling…….4°C.”** | The sentence has been completed. |

Reviewer #2: The manuscript is well written. Its a novel finding in Nepal and authors have used adequate advanced techniques for the laboratory experiment. However, only animal and environmental samples are included and human samples are not included in this study. Therefore the role of novel strain of the organism in human infections needs further research work with inclusive of human samples. Language of the manuscript at same places needs revision

Response: The language has been updated at some places in the manuscript.
